# Supplementary material for: In vitro and in vivo identification of clinically approved drugs that modify ACE2 expression
Source: Mol Syst Biol. 2020 Jul 29;16(7):e9628. doi: 10.15252/msb.20209628 (PMC7390914; doi:10.15252/msb.20209628)
Supplement: Supplementary file 1 — Appendix [file MSB-16-e9628-s001.pdf]

Appendix

In vitro and in vivo identification of clinically-approved drugs that modify ACE2 expression

Appendix for “Systematic cell line-based identification of drugs modifying ACE2 expression”

Sanju Sinha[1,2,*], Kuoyuan Cheng[1,2,*], Alejandro A. Schäffer[1], Kenneth Aldape[3], Eyal Schiff[4], Eytan Ruppin[1†]

1. Cancer Data Science Laboratory (CDSL), National Cancer Institute (NCI), National Institutes of Health (NIH), Bethesda, MD, USA.
2. Center for Bioinformatics and Computational Biology, University of Maryland, College Park, MD, USA.
3. Laboratory of Pathology, National Cancer Institute (NCI), National Institutes of Health (NIH), Bethesda, MD, USA.
4. Department of Obstetrics, Gynecology and Reproductive Sciences, Chaim Sheba Medical Center, Sackler Faculty of Medicine, Tel-Aviv University, Ramat Gan, Tel-Aviv, Israel.

[*] Co-first authors

[†] Corresponding author

Table of Contents

Appendix Note S1. The CMAP data and the selection of cell lines for analysis

- **Appendix Figure S1**

Appendix Note S2. Top drug candidates arising from cancer datasets of lung and kidney across GEO

Note S1. The CMAP data and the selection of cell lines for analysis

The CMAP dataset contains gene expression data of different cells treated by a wide array of different chemical compounds/drugs and measured at different doses and time points. However, not all combinations of cell types, drugs and treatment conditions were available. 24 hours and 10 μM represent the most frequent treatment condition, and therefore, in the main part of our study, we selected samples under this treatment condition for consistency and to result in the largest possible sample size. Besides, since we primarily aimed to choose a set of drugs such that each of the drugs in the set were tested on the same set of cell types for consistency, there exists a trade-off between the number of cell types to include in the analysis and the resulting number of drugs that are tested on all of the selected cell types. Such a trade-off is shown in the Figure S1 below for clinically approved drugs and carcinoma cell lines. It can be seen that there is a drastic decrease in the number of clinically approved drugs available going beyond five cell lines. We decided to include four cell lines as a balanced choice, which resulted in the four (A549, MCF7, PC3 and VCAP) as described in the main text. The focus on carcinoma cell lines is due to their possible higher resemblance to airway epithelial cells (likely one of the major points of viral entry) than some other major cell types available in CMAP, as also explained in the main text.

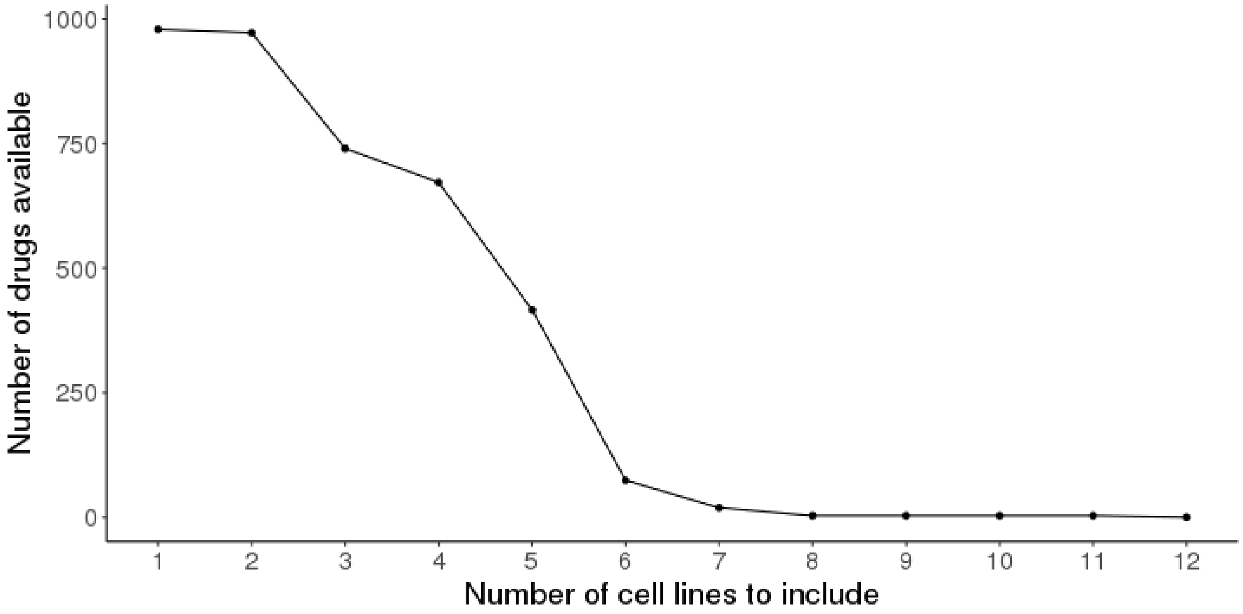

Figure S1. The trade-off in the CMAP dataset between the number of carcinoma cell lines to include in the analysis (X-axis) and the number of available clinically approved drugs that are tested on all of the selected cell lines (Y-axis). Clinically approved drugs were identified from the DrugBank database (explained in main text Methods).

Note S2. Top drug candidates arising from cancer datasets of lung and kidney across GEO

Here, we have summarized the candidate ACE2 drug regulators from the cancer datasets mined for lung from GEO (Figure EV3). One of our candidates is an EGFR inhibitor gefitinib which upregulates ACE2 in three independent datasets in two non-small cell lung carcinoma - HCC4006 and PC9. Another notable example is nicotine which downregulated ACE2 which has been previously also noted in multiple organs (Yue, et al. 2018). This is interesting in context of recent findings of smoking as a risk factor for COVID19 but the evidence is mixed and still developing (Patanavanich and Glantz 2020). Additional candidates include tamoxifen (CMAP logFC=0.21, P=0.002, adjusted P=0.02), an estrogen receptor antagonist, crizotinib (logFC=-0.40, P=0.04, adjusted P=0.07), a receptor tyrosine kinase inhibitor approved for use in a defined subset of non-small cell lung cancer.

References

Yue X, Basting TM, Flanagan TW, Xu J, Lobell TD, Gilpin NW, Gardner JD, Lazartigues E. (2018) Nicotine Downregulates the Compensatory Angiotensin-Converting Enzyme 2/Angiotensin Type 2 Receptor of the Renin–Angiotensin System. *Ann Am Thor Soc.* 2018 Apr;15(Supplement 2):S126-S127

Patanavanich P, Glantz SA. (2020) Smoking Is Associated With COVID-19 Progression: A Meta-analysis, *Nicotine & Tobacco Research,* in press ntaa082, https://doi.org/10.1093/ntr/ntaa082
