# Supplementary material for: In vitro and in vivo identification of clinically approved drugs that modify ACE2 expression
Source: Mol Syst Biol. 2020 Jul 29;16(7):e9628. doi: 10.15252/msb.20209628 (PMC7390914; doi:10.15252/msb.20209628)
Supplement: Supplementary file 2 — Expanded View Figures PDF [file MSB-16-e9628-s002.pdf]

Expanded View Figures

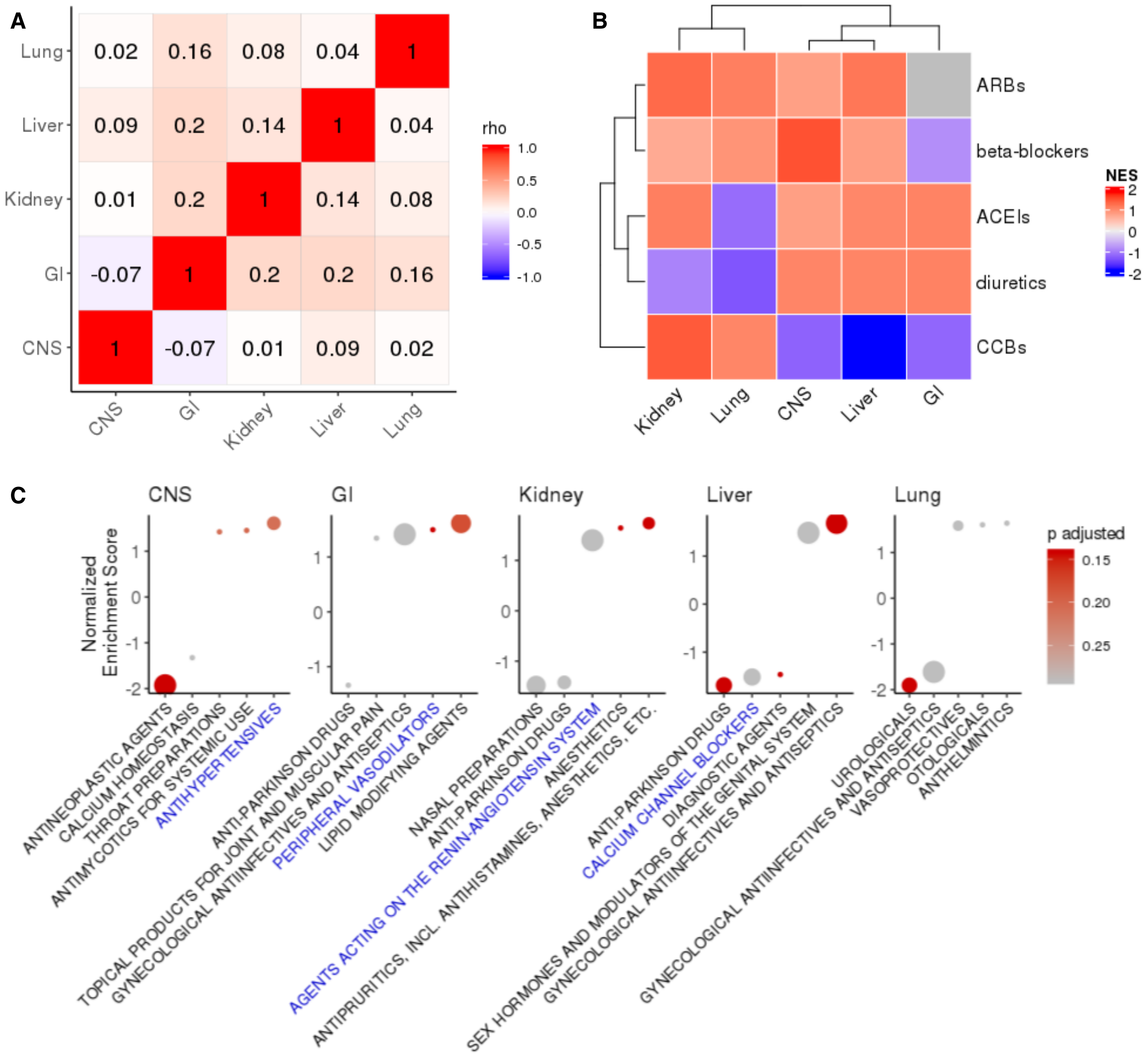

Figure EV1.

© 2020 The Authors

Figure EV1. Comparison of drug-induced *ACE2* differential expression profiles and enrichments of drug classes in *ACE2* modulators in lung, kidney, liver, central nervous system (CNS), and gastrointestinal tract (GI) cells.

A *ACE2* differential expression after drug treatment was analyzed for clinically approved drugs separately in cells from each of the tissues of origin (listed above) in CMAP, as described in the main text. The matrix of Spearman's correlation coefficients for the drug-induced *ACE2* expression fold changes across common drugs between each pair of tissue types are visualized in a heatmap. Spearman's Rho values are labeled in each cell of the heatmap.

B A heatmap visualizing the GSEA normalized enrichment score for the enrichment of each sub-category of antihypertensive drugs in the positive/negative regulators of *ACE2* expression, separately in each tissue type. Positive enrichment score (red) means that the drug class is enriched for drugs upregulating *ACE2* expression, *vice versa* (colored in blue). Missing result is colored gray (for ARBs in GI, since no ARBs are tested in the GI cell line used in the analysis). CCBs, calcium channel blockers; ARBs, angiotensin II type-I receptor blockers; ACEIs, angiotensin-converting enzyme inhibitors.

C Enrichment of each drug class is based on the WHO ATC drug indication in the positive/negative regulators of *ACE2* expression, separately in each tissue type. Top five drug classes (*x*-axis) ranked by significance are visualized for each tissue type (separated into the different panels). The *y*-axis represents the normalized enrichment score as explained above, the dots are colored by level of significance computed with the GSEA method as implemented in the R package fgsea (preprint: Korotkevich *et al*, 2019), and the size of the dots represent the size of the drug class (larger dots correspond to larger drug classes).

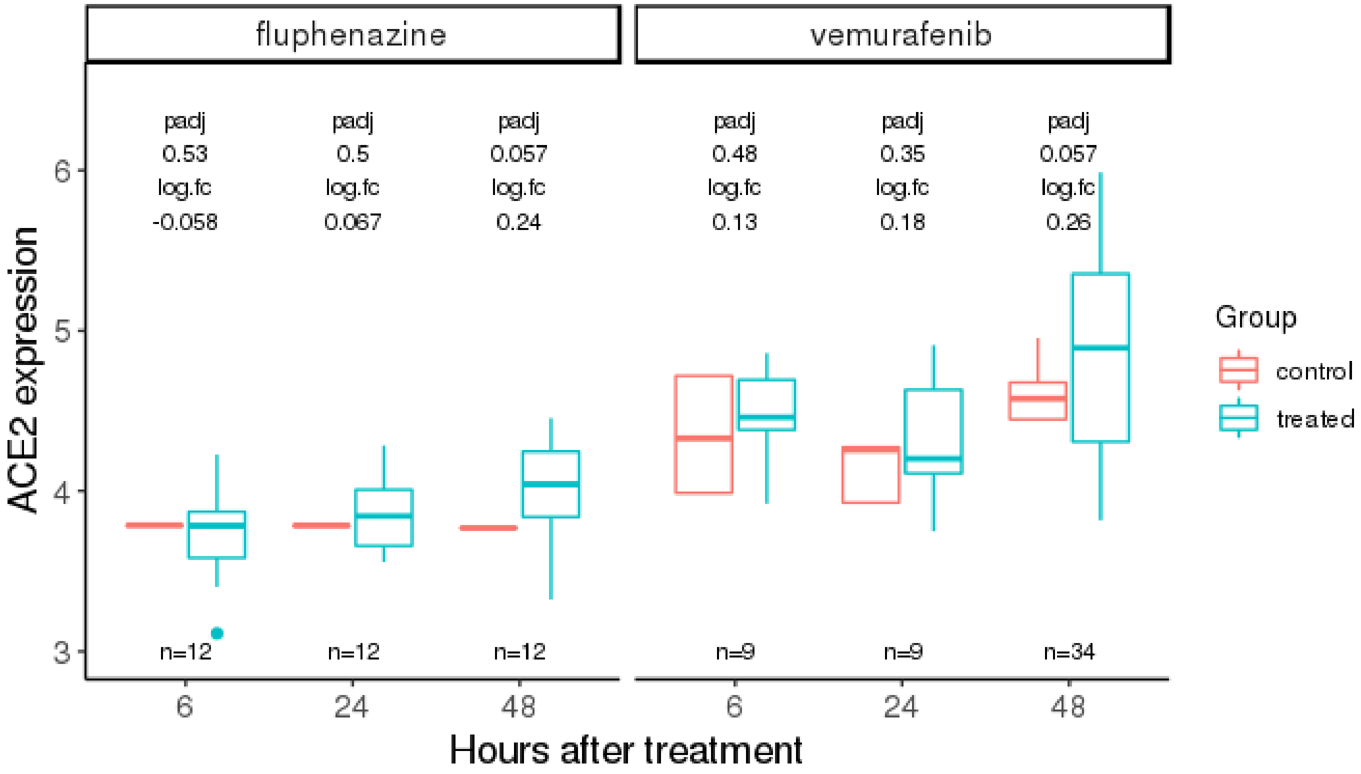

Figure EV2. Time-dependent *ACE2* expression changes induced by fluphenazine and vemurafenib from the CMAP dataset.

Fluphenazine and vemurafenib represent the drugs that significantly modulate *ACE2* expression at the 48-h time point, and for which data are also available at 6- and 24-h time points. The adjusted *P* values (padj) computed using limma (Ritchie *et al*, 2015) and log fold changes (logFC) in treated vs control *ACE2* expressions are labeled above the box plots. We observe a time-dependent increase in the extent of *ACE2* upregulation in treated vs control for both of these drugs. The three *x*-axis values (6, 24, and 48) are placed equidistant not on a linear scale. Here, the center line, box edges, and whiskers in the box plots denote the median, interquartile range, and the rest of the distribution in respective order, except for points that were determined to be outliers using a method that is a function of the interquartile range, as in standard box plots.

© 2020 The Authors

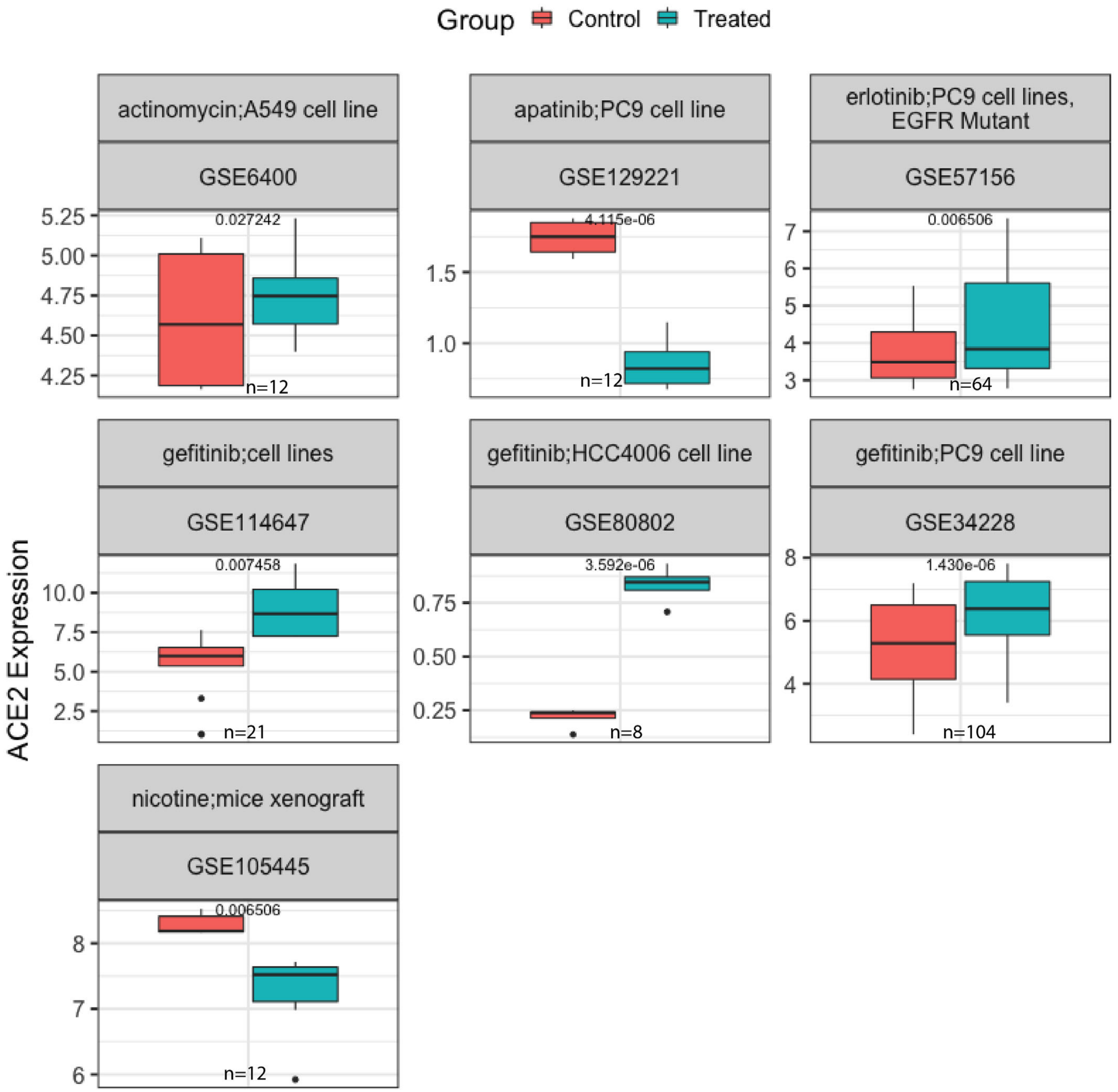

Figure EV3. Significant drug hits with differential *ACE2* expression in control vs treated searching across GEO lung datasets.

Providing the subset of top significant drugs hits from differential analysis of *ACE2* in control vs treated samples from cancer cells with lung tissue of origin, where *ACE2* expression is provided (*y*-axis) in control and treated samples. GEO ID of the respective studies are provided. HBEC, human bronchial epithelial cells. GEO ID of the respective studies are provided. All the *P* values are computed from differential expression analysis using limma (Ritchie *et al*, 2015) (Materials and Methods). Here, the center line, box edges, and whiskers in the box plots denote the median, interquartile range, and the rest of the distribution in the respective order, except for points that were determined to be outliers using a method that is a function of the interquartile range, as in standard box plots.

 © 2020 The Authors
